# Graphene–Cu Nanocomposites Induce Tolerance against *Fusarium oxysporum*, Increase Antioxidant Activity, and Decrease Stress in Tomato Plants

**DOI:** 10.3390/plants12122270

**Published:** 2023-06-11

**Authors:** Diana Cota-Ungson, Yolanda González-García, Gregorio Cadenas-Pliego, Ángel Gabriel Alpuche-Solís, Adalberto Benavides-Mendoza, Antonio Juárez-Maldonado

**Affiliations:** 1 Doctor of Science in Protected Agriculture, Autonomous Agrarian University Antonio Narro, Saltillo 25315, Mexico; green.ungsondiana@gmail.com; 2 Center for Protected Agriculture, Faculty of Agronomy, Autonomous University of Nuevo León, General Escobedo 66050, Mexico; yolanda_glezg@hotmail.com; 3 Applied Chemistry Research Center, Saltillo 25294, Mexico; gregorio.cadenas@ciqa.edu.mx; 4 Institute for Scientific and Technological Research of San Luis Potosi, San Luis Potosí 78216, Mexico; alpuche@ipicyt.edu.mx; 5 Departament of Horticulture, Autonomous Agrarian University Antonio Narro, Saltillo 25315, Mexico; abenmen@gmail.com; 6 Departament of Botany, Autonomous Agrarian University Antonio Narro, Saltillo 25315, Mexico

**Keywords:** antioxidants, antioxidant defense system, biotic stress, nanomaterials, plant pathogens, secondary metabolism, stress tolerance

## Abstract

The tomato crop is susceptible to various types of stress, both biotic and abiotic, which affect the morphology, physiology, biochemistry, and genetic regulation of plants. Among the biotic factors, is the phytopathogen *Fusarium oxysporum* f. sp. *lycopersici* (Fol), which can cause losses of up to 100%. Graphene–Cu nanocomposites have emerged as a potential alternative for pathogen control, thanks to their antimicrobial activity and their ability to induce the activation of the antioxidant defense system in plants. In the present study, the effect of the Graphene–Cu nanocomposites and the functionalization of graphene in the tomato crop inoculated with Fol was evaluated, analyzing their impacts on the antioxidant defense system, the foliar water potential (Ψ_h_), and the efficiency of photosystem II (PSII). The results demonstrated multiple positive effects; in particular, the Graphene–Cu nanocomposite managed to delay the incidence of the “vascular wilt” disease and reduce the severity by 29.0%. This translated into an increase in the content of photosynthetic pigments and an increase in fruit production compared with Fol. In addition, the antioxidant system of the plants was improved, increasing the content of glutathione, flavonoids, and anthocyanins, and the activity of the GPX, PAL, and CAT enzymes. Regarding the impact on the water potential and the efficiency of the PSII, the plants inoculated with Fol and treated with the Graphene–Cu nanocomposite responded better to biotic stress compared with Fol, reducing water potential by up to 31.7% and Fv/Fm levels by 32.0%.

## 1. Introduction

The tomato crop is affected by various types of stress, both biotic and abiotic, which constitutes one of the main limitations for its yield. These stress factors can have a negative impact on various aspects of the plant, including its morphology, physiology, biochemistry, and molecular makeup [1,2]. Among the biotic factors, phytopathogenic microorganisms that are hosted and transmitted through the soil are especially problematic, as they can affect tomato quality and yield [2,3,4]. One of these is the phytopathogen *Fusarium oxysporum* [5], which can persist in soil for long periods of time as dormant chlamydospores, allowing it to survive even in the absence of a host [6]. The pathosystem by tomato (*Solanum lycopersicum* L.) and *Fusarium oxysporum* f. sp. *lycopersici* (Sacc) WC Snyder and HN Hansen (Fol) has been the subject of multiple investigations due to the economic and ecological impact of the fungus derived from its phylogenetic diversity, being distributed by races and special forms that make possible the presence of the disease in many regions of the world [7]; therefore, it presents great challenges for the production of this important crop worldwide [8]. Fol is included among the causative agents of the more than 200 diseases that affect tomato crops [9], including vascular wilt [3]. Fol invades the roots and later colonizes the xylem vessels, avoiding the transport of water, which leads to severe water stress for the plant, whose symptoms manifest as chlorosis in the leaves, beginning in the lower third of one side only in leaflets, accompanied by vascular discoloration, wilting, and finally plant death [10,11] causing losses up to 100% [8].

In response to the pressure exerted by biotic and abiotic factors, plants in their evolution have developed different defense systems which are associated with a type of response and are classified into two categories: passive or pre-formed defense systems (pre-existing) and another kind of active or induced defense [12,13]. The passive defense system is closely associated with inherent structural features of the plant, such as cuticles, trichomes, waxes, and secondary metabolites [14]. On the other hand, the active or induced defense mechanism is generally related to the endogenous production or exogenous application of compounds known as elicitors. An elicitor is a chemical substance or compound that, when applied in small amounts to plants, has the ability to trigger or enhance the production of a particular compound that is crucial for the plant to adapt to stress conditions [15]. Elicitors are molecules of synthetic or organic origin and, despite the fact that they are very varied, their purpose is the same and they are classified based on their characteristics, according to their origin as endogenous and exogenous or according to their nature as biotic or abiotic [16]. 

Nanomaterials have been reported as elicitors [17,18], which are a simple method to increase the production of secondary metabolites in plant cells and tissues [19]. Recently, the resistance of pests/pathogens has increased due to the excessive and inappropriate use of conventional agrochemicals, requiring higher doses to be controlled. Compared with traditional products and methods, nanotechnology is a promising new approach for plant disease management with the aim of inducing biostimulation with better efficacy, lower input requirements, and lower ecotoxicity [20]. 

Graphene is a two-dimensional structure of carbon atoms with sp2 hybridization, arranged in a hexagonal arrangement; it also has extraordinary electronic properties and the ability to transport electrons [21]. Carbon nanomaterials (CNMs) such as functionalized graphene are an emerging class of novel materials that may exhibit considerable antimicrobial activity [22], while copper nanoparticles (Cu-NPs) are well-known for their antibacterial effects, which penetrate cells and cause cell death. However, pure Cu-NPs tend to agglomerate, which prevents their antimicrobial action [23]. For this reason, the combination of these two materials at the atomic or nanometric level (Graphene–Cu) allows each of the materials to complement each other to have new and improved functions and properties that ensure a greater interrelation between the constituent materials [24]. The effect of nanomaterials as elicitors will depend on the physical and chemical characteristics of the nanomaterials, concentrations, the biological species with which they interact, the form and route of application, and the biological surfaces where the interaction occurs [17,25]. In addition, CNMs as biostimulants increase the productivity of agricultural crops by increasing the absorption and retention capacity of water in cells, improving the metabolic and physiological activity of plants by developing cellular defense mechanisms [26]. Therefore, they have been mentioned as a fundamental tool to control the adverse effects of abiotic stress [27].

Based on the above, the present work aims to evaluate the effect of Graphene–Cu nanocomposites and functionalized graphene NMs on the growth and development of tomato plants affected by the “vascular wilt” disease and stimulate tolerance against Fol, by analyzing physiological and metabolic changes in response to biotic stress.

## 2. Results

### 2.1. Incidence and Severity 

**Figure 1:**
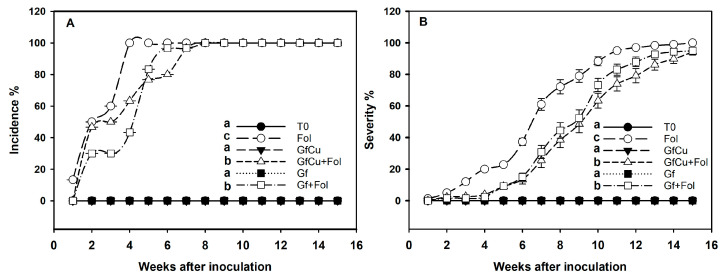
Incidence (**A**) and severity (**B**) of *Fusarium oxysporum* f. sp. *lycopersici* on the tomato plants, starting 2 weeks after the inoculation. T0: Negative control, Fol: Positive control inoculated with Fol, GfCu: Graphene–Cu nanocomposites, GfCu + Fol: Graphene–Cu nanocomposites + Fol, Gf: Functionalized graphene, and Gf + Fol: Functionalized graphene + Fol. Different letters indicate significant differences according to the Hotelling Test (*p* < 0.05).

Highly significant differences were observed between the treatments evaluated for the control of vascular wilt disease in tomatoes. The incidence of the disease was presented in a statistically significant way in the Fol treatment, reaching 100% at 4 weeks after inoculation, while in the treatments with Graphene–Cu nanocomposites and functionalized graphene NMs (GfCu + Fol and Gf + Fol, respectively) the incidence of 100% was reached at 8 weeks after inoculation (Figure 1A).

**Figure 2:**
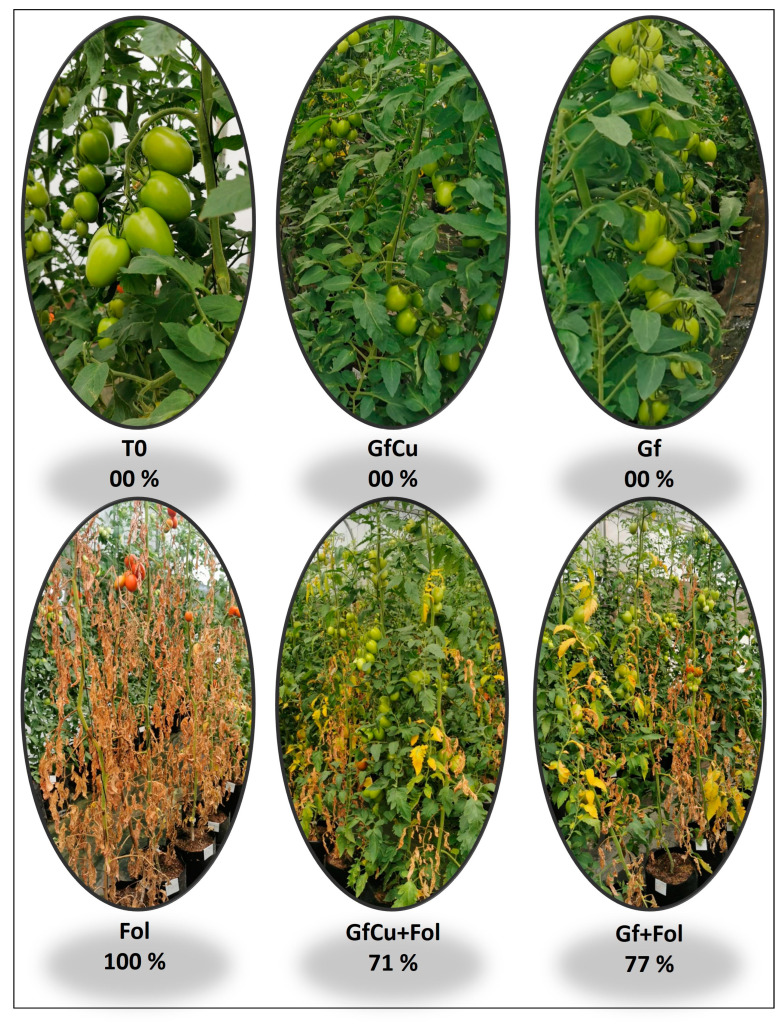
State of development and severity of the tomato plants 10 weeks after transplanting. T0: Negative control, Fol: Positive control inoculated with Fol, GfCu: Graphene–Cu nanocomposites, GfCu + Fol: Graphene–Cu nanocomposites + Fol, Gf: Functionalized graphene, and Gf + Fol: Functionalized graphene + Fol.

On the other hand, it was found that the application of Graphene–Cu nanocomposites and functionalized graphene NMs significantly decreased the severity of the disease by 29.0% and 23.0%, respectively, compared with that of the positive control inoculated with Fol (Figure 1B and Figure 2). 

### 2.2. Agronomical Parameters

**Figure 3:**
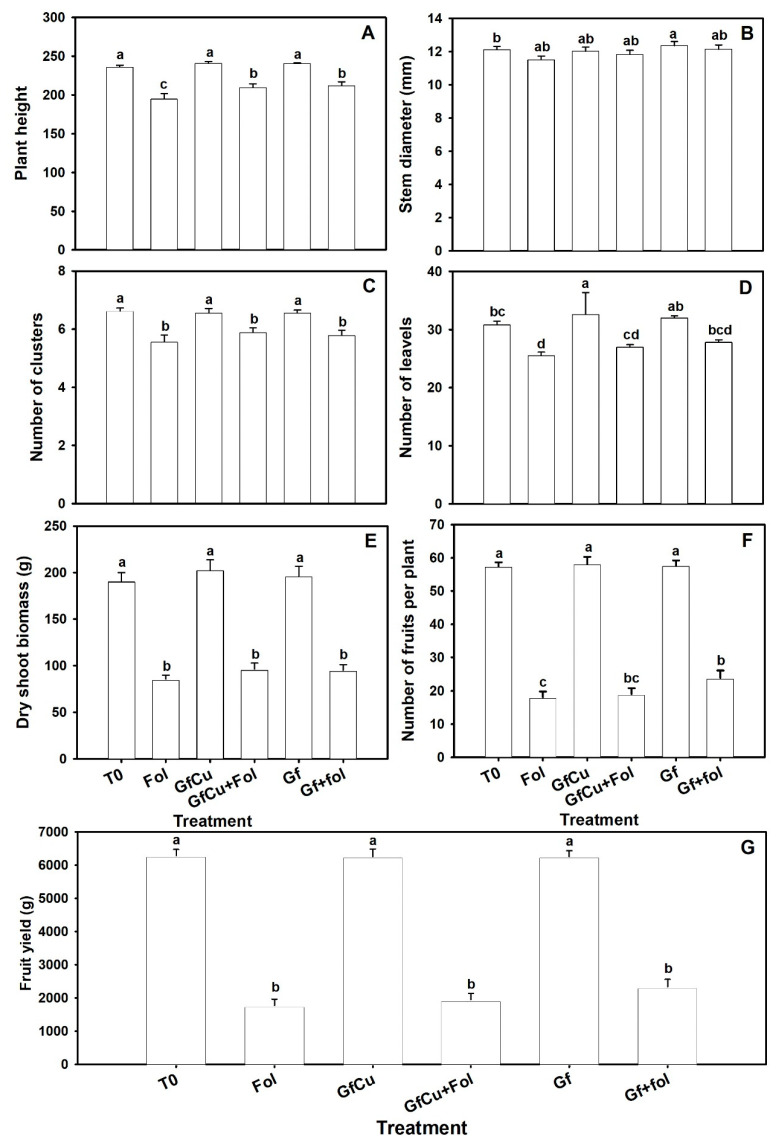
Plant height (**A**), stem diameter (**B**), number of clusters (**C**), number of leaves (**D**), dry biomass (**E**), number of fruits per plant (**F**), and fruit yield per plant (**G**) of the tomato crop. T0: Negative control, Fol: Positive control inoculated with Fol, GfCu: Graphene–Cu nanocomposites, GfCu + Fol: Graphene–Cu nanocomposites + Fol, Gf: Functionalized graphene, and Gf + Fol: Functionalized graphene + Fol. Different letters indicate significant differences according to the Fisher’s least significant difference test (*p* < 0.05).

Significant differences were observed between the treatments inoculated with Fol. The Fol treatment presented a decrease in the height of tomato plants of 17.4% compared with the T0 treatment, while in the GfCu + Fol and Gf + Fol treatments the decrease was 11.3% and 10.3%, respectively (Figure 3A). An increase in stem diameter of 2.0% was observed in the Gf treatment compared with the negative control (T0), which showed a statistically significant difference (Figure 3B). Compared with the Fol treatment, the GfCu + Fol and Gf + Fol treatments showed an increase in stem diameter of 2.8% and 5.5%, respectively.

The results indicate that there were no significant differences in the number of clusters between the healthy treatments and the treatments inoculated with Fol. All the inoculated treatments had a number of clusters similar to the Fol treatment and the T0 negative control (Figure 3C). Regarding the number of leaves, an increase of 5.7% was observed in the GfCu treatment compared with the negative control (T0); this difference was statistically significant. On the other hand, the application of NMs resulted in a decrease in foliage in plants inoculated with Fol. In particular, the GfCu + Fol and Gf + Fol treatments reduced foliage by 12.3% and 9.7%, respectively, while the Fol treatment had a reduction of 17.1% compared with the negative control (T0) (Figure 3D).

In dry biomass, no significant differences were observed between the applied treatments and the negative control in healthy plants, nor were any statistically significant differences found between the inoculated treatments and the positive control (Fol) (Figure 3E). In plants inoculated with Fol, the application of CNMs had a positive effect on the number of fruits. In the GfCu + Fol treatment, an increase of 5.0% in the number of fruits was observed, while in the Gf + Fol treatment an increase of 32.0% was obtained compared with the positive control (Fol) (Figure 3F). This increase in the number of fruits was also reflected in a yield increase of 9.9% and 31.6% for the GfCu + Fol and Gf + Fol treatments, respectively, compared with the Fol treatment, despite not observing significant differences between treatments (Figure 3G).

### 2.3. Physiological Variables 

**Figure 4:**
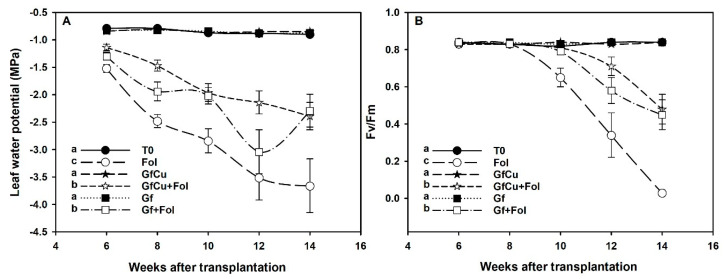
Foliar water potential (**A**) and Fv/Fm ratio (efficiency of photosystem II) in the tomato crop (**B**). T0: Negative control, Fol: Positive control inoculated with Fol, GfCu: Graphene–Cu nanocomposites, GfCu + Fol: Graphene–Cu nanocomposites + Fol, Gf: Functionalized graphene, and Gf + Fol: Functionalized graphene + Fol. Different letters indicate significant differences according to the Fisher’s least significant difference test (*p* < 0.05).

Significant differences were observed between the treatments in the foliar water potential during the development of the tomato crop in healthy plants. While the values remained between 0.5 and −1.0 (MPa) in healthy plants, the inoculated positive control treatment (Fol) showed a significant decrease until reaching a value of −3.5 (MPa) at the end of the crop, with a mean of −2.67 (MPa). On the other hand, the GfCu + Fol and Gf + Fol treatments registered an average of −1.85 and −1.98 (MPa), respectively. In particular, the application of Graphene–Cu nanocomposites and functionalized graphene NMs in plants inoculated with Fol significantly decreased water potential by 31.7% and 25.9%, respectively, compared with the positive control inoculated (Fol) during the mornings (Figure 4A).

Through the analysis of the photochemical activity of PSII, significant differences were found between the evaluated treatments. The results showed that both the GfCu + Fol and Gf + Fol treatments were able to improve the Fv/Fm ratio by 32.0% and 27.6%, respectively, compared with the positive control inoculated with Fol (Figure 4B). These findings suggest that the application of Graphene–Cu nanocomposites and functionalized graphene NMs could contribute to an improvement in photochemical activity in plants affected by this disease.

### 2.4. Photosynthetic Pigments

**Figure 5:**
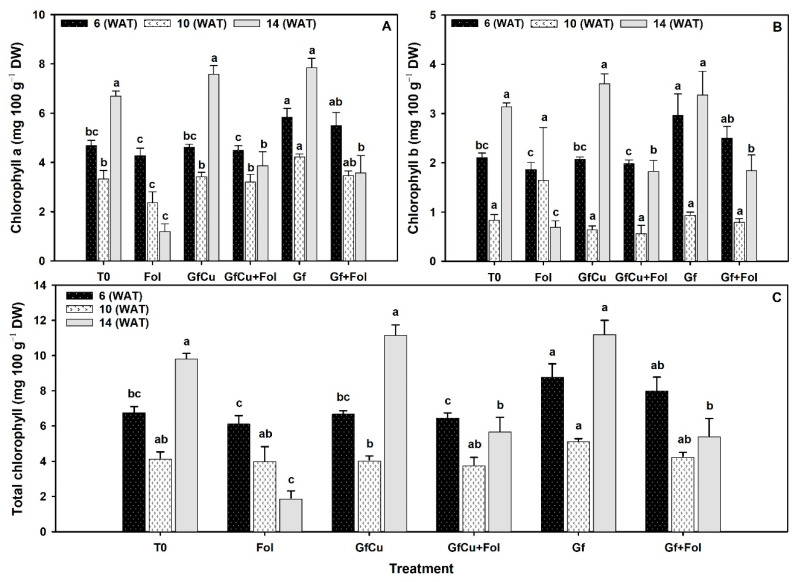
Chlorophyll a (**A**), chlorophyll b (**B**), and total chlorophyll (**C**). Evaluation one at 6 WAT, evaluation two at 10 WAT, and evaluation three at 14 WAT. T0: Negative control, Fol: Positive control inoculated with Fol, GfCu: Graphene–Cu nanocomposites, GfCu + Fol: Graphene–Cu nanocomposites + Fol, Gf: Functionalized graphene, and Gf + Fol: Functionalized graphene + Fol. Different letters indicate significant differences according to the Fisher’s least significant difference test (*p* < 0.05).

Differences were observed between treatments in the content of photosynthetic pigments, including chlorophyll a, b, and total. In samplings one and two, the Gf treatment showed the highest chlorophyll a content, followed by the Gf + Fol treatment, while the Fol treatment presented the lowest chlorophyll a content and differed significantly from the rest of the treatments. In sampling three, the Gf treatment showed the highest chlorophyll a content, with an increase of 17.1% compared with the negative control (T0), followed by the GfCu + Fol treatment, while the Fol treatment showed the lowest chlorophyll a content (Figure 5A). 

Regarding the content of chlorophyll b, in sampling one the highest content was observed with the Gf treatment and the lowest with the Fol treatment, while in sampling two the Fol treatment showed the highest content, but did not differ significantly from the rest of the treatments. In sampling three, the lowest chlorophyll b content was observed in the Fol treatment, which differed significantly from the rest of the treatments (Figure 5B).

In relation to the total chlorophyll content, in sampling one the Gf treatment showed up to 29.4% more compared with the negative control (T0) and differed significantly from the rest of the treatments. In sampling two, the total chlorophyll content was higher in the Gf treatment, with 23.4% more compared with the negative control (T0), followed by the Gf + Fol treatment. In sampling three, the Gf treatment showed the highest total chlorophyll content with 14.1% more than the negative control (T0), followed by the GfCu + Fol treatment (Figure 5C).

### 2.5. Antioxidant Compounds

**Figure 6:**
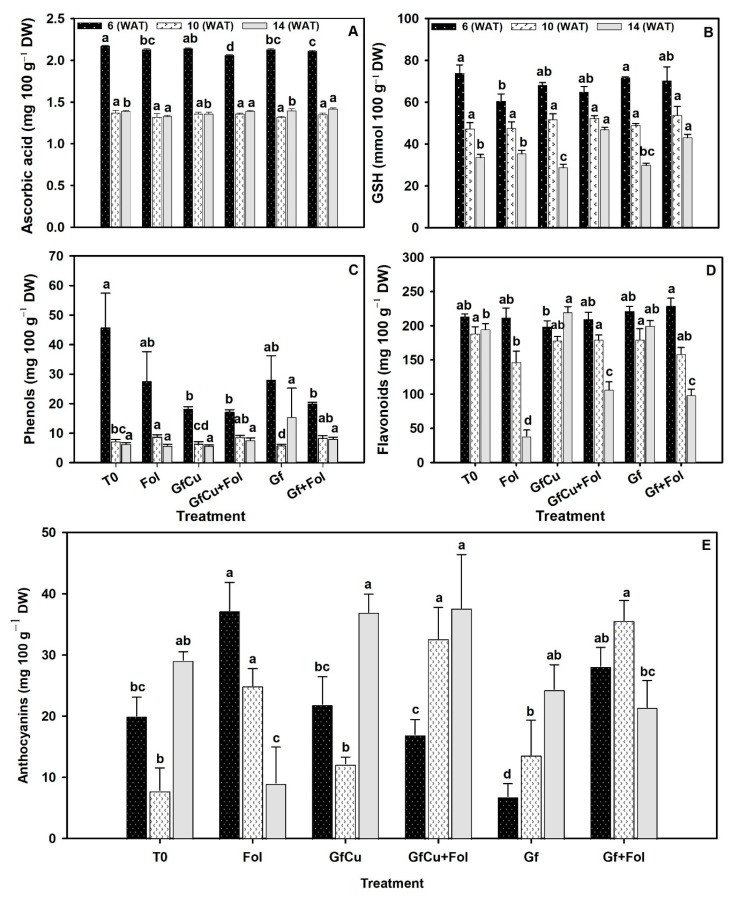
Ascorbic acid (**A**), glutathione (GSH) (**B**), phenols (**C**), flavonoids (**D**), and anthocyanins (**E**) in tomato crop leaves. Evaluation one at 6 WAT, evaluation two at 10 WAT, and evaluation three at 14 WAT. T0: Negative control, Fol: Positive control inoculated with Fol, GfCu: Graphene–Cu nanocomposites, GfCu + Fol: Graphene–Cu nanocomposites + Fol, Gf: Functionalized graphene, and Gf + Fol: Functionalized graphene + Fol. Different letters indicate significant differences according to the Fisher’s least significant difference test (*p* < 0.05).

The results obtained in the three samplings indicated variations in the content of ascorbic acid and glutathione in the different treatments evaluated. In the first sampling, the GfCu + Fol treatment showed the lowest ascorbic acid content and significant differences with the other treatments. However, in the second sampling, no statistically significant differences were observed between the evaluated treatments (Figure 6A). In the third sampling, the Gf treatment presented the highest content of ascorbic acid. On the other hand, in terms of glutathione content, an increase of 16.3% was found in the Gf + Fol treatment in the first sampling compared with the inoculated positive control (Fol). In the second sampling, no significant differences were observed between treatments (Figure 6B). Finally, in the third sampling, it was observed that the GfCu + Fol and Gf + Fol treatments presented the highest glutathione content, with an increase of 32.5% and 21.8%, respectively, compared with the inoculated positive control (Fol). 

In the content of phenols, no significant differences were found between treatments in samplings one and two; however, it was interesting to note that T0 presented the highest concentration of phenols in the first sampling (Figure 6C). In sampling three, an increase in the phenol content was observed with the Gf treatment, increasing up to 140.0% compared with the negative control (T0), followed by the Gf + Fol treatment, which showed an increase of 38.4% compared with the inoculated positive control (Fol).

In the flavonoid content, different results were observed in the three samplings carried out. In the first sampling, the Gf + Fol and Gf treatments showed an increase of 7.4% and 3.7%, respectively, compared with the negative control (T0). However, in the second sampling, it was found that the T0 treatment presented the highest flavonoid content, while the Fol treatment had the lowest. Finally, in the third sampling, it was evidenced that the GfCu treatment presented the highest flavonoid content, while the lowest was observed in the Fol treatment. It should be noted that the GfCu + Fol and Gf + Fol treatments showed a significant increase of 22.3% and 8.0%, respectively, compared with the inoculated positive control (Fol) in sampling two, and even increased to 174.5% and 154.6% of flavonoids, respectively, in sampling three. In addition, these results were statistically different from the rest of the treatments (Figure 6D).

Significant differences were found in all samplings for anthocyanins. In sampling one, the Fol treatment presented the highest anthocyanin content, while in sampling two an increase in anthocyanin content was observed in the GfCu + Fol and Gf + Fol treatments, with the T0 treatment presenting the lowest content. In sampling three, it was found that the GfCu treatment presented the highest anthocyanin content, with an increase of 26.9% compared with the negative control (T0) (Figure 6E). It is important to highlight that the GfCu + Fol and Gf + Fol treatments showed significant increases in anthocyanin content of 313.5% and 153.5%, respectively, compared with the positive control (Fol).

### 2.6. Enzymatic Activity in Tomato Leaves

**Figure 7:**
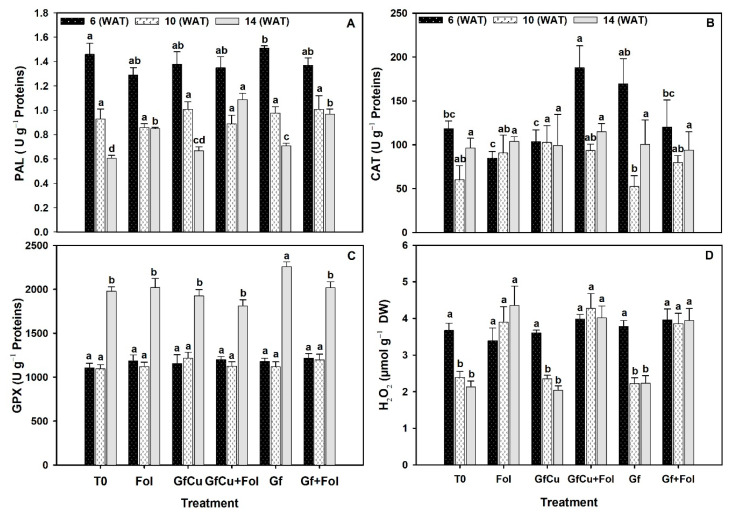
Enzymatic activity of phenylalanine ammonium lyase (PAL) (**A**), catalase (CAT) (**B**), glutathione peroxidase (GPX) (**C**), and hydrogen peroxide (H_2_O_2_) (**D**) in tomato crop leaves. Evaluation one at 6 WAT, evaluation two at 10 WAT, and evaluation three at 14 WAT. T0: Negative control, Fol: Positive control inoculated with Fol, GfCu: Graphene–Cu nanocomposites, GfCu + Fol: Graphene–Cu nanocomposites + Fol, Gf: Functionalized graphene, and Gf + Fol: Functionalized graphene + Fol. Different letters indicate significant differences according to the Fisher’s least significant difference test (*p* < 0.05).

In relation to the activity of the enzyme phenylalanine ammonium lyase (PAL), notable differences were observed between the evaluated treatments. In particular, the Gf treatment showed the highest PAL content in the first analysis, while the Fol treatment presented the lowest. In the second sampling, no significant differences were found between the treatments. In the third sampling, the GfCu + Fol treatment stood out by presenting an increase of 28.2% in the PAL content compared with the inoculated positive control (Fol), and this result was statistically different from the rest of the evaluated treatments (Figure 7A).

During the first sampling, a notable increase in the catalase content was observed in the GfCu + Fol treatment, which showed a significant increase of 120.8% compared with the inoculated positive control (Fol), which, in turn, presented the least amount of this enzyme. In addition, the Gf treatment also showed a 43.0% increase compared with the negative control (T0). However, in the second sampling, it was observed that the Gf treatment presented the lowest catalase content. Finally, in the third sampling, although there were no statistically significant differences between the evaluated treatments, it was observed that the GfCu + Fol treatment again presented the highest catalase content (Figure 7B).

During the first and second samplings, no statistically significant differences were found between the evaluated treatments in terms of glutathione peroxidase enzyme activity (Figure 7C). However, in the third sampling, an increase of 13.9% in the enzyme activity was observed in the Gf treatment compared with the negative control (T0), with this treatment being statistically different from the rest of the evaluated treatments.

The results of the hydrogen peroxide content indicate that in sampling one there were no statistically significant differences between the treatments (Figure 7D). In sampling two, the treatments with Fol showed similar values, with an increase observed in the GfCu + Fol treatment and also in the Fol treatment in the third sampling.

## 3. Discussion

### 3.1. Impact of F. oxysporum f. sp. lycopersici and Nanocomposites on Plant Growth and Development 

The disease “vascular wilt” in tomatoes is one of the most important diseases that affects the tomato crop, since it can cause significant losses in the production and quality of the fruits. Furthermore, it can affect the ability of the plant to absorb and transport nutrients and water, which can have a negative impact on its growth and development. To cope with the adverse effects of abiotic and biotic stress, the application of nanomaterials as biostimulants has been considered as a potential solution. The direct application to the substrate of the Graphene–Cu nanocomposite and functionalized graphene showed a decrease in the severity of the “vascular wilt” disease in tomatoes (Figure 1B and Figure 2). This reduction in biotic stress is highly related to the uptake and translocation of the nanocomposite within the plant system [28]. During the first stage of the process of absorption of nanomaterials by the plant, they adhere to the root surface and begin to interact with different compounds secreted by the root. The nanomaterials can then interact with structures such as the epidermis, cortex, Caspary strips, and endodermis before continuing their journey to other plant tissues and organs, via the apoplastic or symplastic pathway [29]. Scanning Electron Microscopy (SEM) images demonstrated that the crucial site for graphene entry into root cells and its subsequent translocation through the symplastic pathway is the apex of the growing root hair. At this point, the primary cell wall is significantly thinner, making it easier for graphene nanoparticles to enter [30]. Once inside, NMs interact with plants at the cellular and subcellular level, promoting changes in morphological and physiological states [31].

In addition, carbon-based nanomaterials (CNMs) are known for their antimicrobial activity, especially in nanocomposite forms suitable for protecting crops against the effects of pathogens [22,32]. Prominent examples of CNMs with effective antimicrobial nanoactivity against different plant pathogens are carbon nanotubes, fullerene, and graphene [33]. In addition, it has been found that copper nanoparticles also have an enhanced effect on plant growth and prevention of Fusarium wilt, while promoting tomato plant growth and raising chlorophyll content; this is because Cu-NPs effectively deliver copper as a micronutrient to plants [34]. Regarding the mechanisms of action of the CNMs, direct contact is one of the most relevant, since it can cause rupture of the cell wall and the cytoplasmic membrane, alterations in the fluidity of the membrane, oxidative stress, inhibition of enzymes, and reduced transcription of key genes [35]. Copper nanoparticles have also demonstrated antimicrobial efficacy due to increased production of reactive oxygen species, which cause lipid peroxidation, membrane disintegration, and the emergence of genomic DNA from pathogens such as *Xanthomonas oryzae* pv. *oryzae* [36].

CNMs have been shown to increase tolerance to various diseases. For example, CNMs inhibit the growth of *Fusarium verticillioides* in vitro and in vivo, which causes corn stalk rot, as well as increasing the level of carotenoid, anthocyanin, and chlorophyll pigments [37]. In tomato plants infested with *Xanthomnas euvesicatoria* 7 days after inoculation, they indicated a significantly lower disease severity (15.6%) when treated with reduced oxide nanocomposite with copper and silver (rGO-Cu-Ag) compared to the positive control, which obtained a severity of 77.1%. Furthermore, tomato plants showed significantly reduced symptoms when treated with a concentration of 500 µg mL^−1^ [38]. González-García et al. [39] indicated that the application of CNMs significantly reduced the severity of *F. oxysporum* and, consequently, increased the yield of tomato fruits. There was also an increase in the levels of photosynthetic pigments, ascorbic acid, and flavonoids, as well as in the activity of antioxidant enzymes in the leaves of plants inoculated with the pathogen. These results suggest that the use of CNMs could be a viable option to control diseases such as *F. oxysporum* in tomato crops. Foliar application of bio-CuNP enhanced copper accumulation in plants, promoting growth and photosynthesis of watermelons, and suppressed bacterial spots on watermelons caused by *Acidovorax citrull* by activating an immune response in plants [40]. 

Due to biotic stress, plant growth was affected by an imbalance that occurs in hormonal signaling pathways. Fol decreased the percentage of height and foliage in tomato plants, while the application of NMs improved growth in plants compared with Fol. In addition, the GfCu + Fol and Gf + Fol treatments increased the number of fruits compared with Fol, also increasing yield. In fact, the effect of Fol on the growth parameter of the tomato plant has been verified in several studies, demonstrating that there is a significant reduction in plant height, leaf area, and fresh and dry biomass [41]. It is important to highlight that the nanomaterials greatly promoted the growth, stems, leaves, and fruits of healthy and Fol-inoculated tomato plants (Figure 3).

The positive growth was due to the fact that CNMs could act as signaling molecules to stimulate the biosynthesis of plant hormones in plants. Xiong et al. [42] observed that the application of fullerene nanoparticles in concentrations of 1–100 mg L^−1^ resulted in a significant increase in abscisic acid (ABA) levels in *Brassica napus* leaves. These nanoparticles also induced ABA biosynthesis by reducing the expression of the catabolic ABA gene *CYP707A3*, which improved drought tolerance in *B. napus* seedlings. In addition, the application of fullerene via foliar route increased the antioxidant capacity of the plant, which allowed a collective detoxification of reactive oxygen species (ROS). Exposure to single-walled nanotubes (SWNTs) in tomato plants significantly increased salicylic acid (SA) content [43]. Guo et al. [44] observed that the application of graphene oxide (GO) at concentrations of 50 mg L^−1^ and 100 mg L^−1^ in tomato plants resulted in a significant increase in root auxin content. In this context, nanomaterials have the ability to increase the resistance of host plants to phytopathogens, which can help prevent the development of diseases in plants and contribute to better performance and growth of plants; in time, this can increase production and improve the quality of crops [45]. 

### 3.2. Impact of F. oxysporum f. sp. lycopersici and Nanocomposites on Water Potential and Fluorescence of Chlorophyll a 

Fol penetrates the plant through the roots and moves into the vascular bundles [46]. Once inside the xylem, the microconidia and hyphae of the fungus, together with the secretions of polysaccharides and pectinolytic enzymes, decrease the water potential in the leaves and stems of the plant [11]. Exposure to Fol decreased water potential by 215.3% compared with the negative control (Figure 4A). However, the application of GfCu and Gf in plants inoculated with Fol reduced water stress by 31.7 and 25.9%, respectively, compared with the positive control (Fol). This indicates that Fol directly affects the water absorption capacity of tomato plants, causing water stress as a consequence. The reduction in water stress was due to the antimicrobial action of graphene, which causes physical damage to the microorganism’s membranes by coming into direct contact with their sharp edges and destructively extracting lipid molecules [47].

Another strategy to mitigate the effects of water stress caused by Fol is the interaction of graphene with plant roots. According to [48], when plant roots are exposed to graphene, there is an increase in the H_2_O, NH_4_^+^, and K^+^ contents of the soil due to hydrogen bonding, electrostatic attraction, and cation–π interactions. This, in turn, leads to the accumulation of H_2_O, NH_4_^+^, and K^+^ on the graphene surface. Graphene has also been reported to have the ability to prevent the evaporation of water from soil thanks to its oxygen-containing hydrophilic functional groups [49]. In addition, it has been reported that carbon nanomaterials can alter the expression of proteins crucial for stress signaling and water transport in plants, such as the aquaporins PIP1s and PIP2s, which are considered crucial membrane proteins for the transport of water [50,51].

On the other hand, it has been shown that the fluorescence of chlorophyll a can be used as an indicator of stress in plants [52]. A decrease in fluorescence parameters occurs gradually as Fusarium wilt develops [53]. In this work, a reduction in Fv/Fm values of 34% was observed at 90 days post-inoculation in Fol compared with the negative control (T0). The GfCu + Fol and Gf + Fol treatments significantly reduced Fv/Fm levels by 32.0% and 27.6%, respectively, compared with the positive control (Fol) (Figure 4B). This is because the graphene accumulated in the leaves and passively transported to the chloroplasts facilitates the process of electron transfer from the PSII to the thylakoids and protects the PSII against photobleaching by acting as a scavenger of reactive oxygen species [54]. In addition, it increases the level of enzymes related to the defense system and improves the assimilation of N in chloroplasts [55]. It has also been discovered that, upon interaction with nanomaterials, plant organelles can acquire new and improved functions. Lu et al. [54] found that about 44% and 29% of the graphene accumulated in rice leaves was passively transported to chloroplasts and thylakoids, respectively. This process considerably improved the fluorescence intensity of the chloroplasts and increased the production of adenosine triphosphate by 2.4 times. 

Fullerene (FLN) derivatives, such as carbon nanostructures, have been studied and their ability to protect the photosynthetic apparatus of *Zea mays* and preserve photochemistry in photosystems (PSI-PSII) has been demonstrated, as well as preventing damage in the energy flux and fluorescence transients of chlorophyll under Co stress [55]. Sulfonated graphene oxide (SGO) has been shown to improve the tolerance of nitrate (NS)- and ammonium (AS)-stressed wheat chloroplasts by enhancing potential photochemical efficiency and chlorophyll fluorescence, inducing core protein expression reactions related to photosystems, regulate the AsA-GSH cycle, and prevent the accumulation of radicals produced by NS and AS [56]. Regarding the phytotoxicity of carbon-based nanomaterials, reduced graphene oxide (RGO) and graphene oxide (GO) have been found to mainly impair photosynthesis in *Brassica napus* L., decreasing chlorophyll content and Rubisco activity, and altering the structure of the chloroplast, respectively. In contrast, amine-functionalized graphene (G-NH_2_) has not shown significant toxicity at concentrations of 10–1000 mg L^−1^ [57]. In general, carbon-based nanomaterials have been shown to improve chloroplast tolerance and photosynthetic efficiency in plants under different types of stress, including infection by phytopathogenic fungi such as Fusarium.

### 3.3. Impact of F. oxysporum f. sp. lycopersici and Nanocomposites on Photosynthetic Pigments

Exposure to Fol resulted in a 41.8% decrease in total chlorophyll content compared with the negative control (Figure 5). This reduction in the chlorophyll content and changes in its proportion represent a physiological alteration in plants [58]. It has been observed that water stress is one of the main factors that contributes to the decrease in chlorophylls, due to the closure of stomata and the accumulation of reactive oxygen species (ROS), which can cause irreversible damage to chloroplasts and a decrease in chlorophylls in photosynthetic efficiency [3]. On the other hand, the use of nanomaterials in plants produces changes in the photosynthesis process, photochemical reactions, quantum yield, and photosynthetic pigments [59]. 

Several studies have shown that the application of carbon-based nanomaterials, such as carbon nanotubes, graphene oxide, polyhydroxyfullerene, as well as fullerol, can improve chlorophyll content and photosynthetic activity in plants subjected to abiotic or biotic stress [60,61,62,63]. In particular, in this study it was observed that the use of GfCu and Gf in healthy plants produced an increase of 5.5% and 21.0%, respectively, in the content of photosynthetic pigments. In addition, in plants inoculated and treated with graphene (GfCu + Fol and Gf + Fol), an increase of 32.2% and 46.4%, respectively, was obtained compared with the Fol treatment (Figure 5C). This is because the presence of nanomaterials in the chloroplast can have multiple positive effects, such as the induction of chlorophylls and carotenoids. In addition, it acts as a carbon source that facilitates fixation as carbon and increases the speed of electron transport, thus inducing an improvement in photosynthesis [64].

### 3.4. Impact of F. oxysporum f. sp. lycopersici and Nanocomposites on Antioxidant Defense Systems

In recent years, ROS have been shown to play an important role as signaling molecules in plants, participating in different processes such as growth and development [65]. In this context, nanomaterials can act as a “stressor” in plants, triggering the production of antioxidants as a protective response [66]. Nanomaterials interact with various plant cell structures, such as the cell wall, cell membrane, different organelles, and even the nucleus, and this can induce a series of responses [67]. This interaction between nanomaterials and plants can induce a hormesis effect [68], which can increase tolerance to different types of biotic or abiotic stress [27,69]. Therefore, the effect of nanomaterials on the response of plants to infection by Fol, a fungus that produces mycotoxins that stimulate excessive ROS production at the cellular level, which can cause oxidative damage in plants [3].

To prevent the spread of oxidative stress and protect themselves from its harmful consequences, plant cells activate antioxidant systems. These systems have the capacity to transform free radicals (oxidants) generated by ROS into less toxic molecules. Among the most prominent antioxidant compounds are those of an enzymatic nature, such as superoxide dismutase, peroxidases, and catalase, among others [70]. In the present study, it was observed that GfCu + Fol and Gf + Fol treatments increased the amount of secondary metabolites such as glutathione (GSH), flavonoids, and anthocyanins compared with Fol (Figure 6B,D,E). This increase was detected on average during the three samplings carried out and may be due to the increase in these metabolites, which decreased the severity of the “vascular wilt” disease (Figure 1B and Figure 2). The glutathione (GSH) molecule is made up of three amino acids, L-cysteine, L-glutamic acid, and glycine, and plays an important antioxidant role [71]. In addition to its abundance, GSH has a fundamental role in improving the tolerance of plants to biotic and abiotic stress, since its main function is to counteract free radicals and detoxify reactive oxygen species (ROS) that are generated in unfavorable conditions. It is also important to note that GSH acts as a cellular signal in plant stress signaling pathways, directly or in conjunction with the glutaredoxin and thioredoxin systems [72]. Flavonoids are responsible for the pigmentation of flowers, leaves, and fruits, and have an important function in the protection against UV radiation and diseases [73]. Anthocyanins can prevent lipid peroxidation and act on ROS in the vacuole by increasing their content in response to various stress conditions [74]. Ozfidan-Konakci et al. [75] reported that in cobalt-stressed *Zea mays* seedlings exposed to concentrations of 100 and 250 mg L^−1^ of fullerene, hydrogen peroxide (H_2_O_2_) was eliminated through enzymes and non-enzymes related to the AsA-GSH cycle, which preserved ascorbate (AsA) conversion, as well as the redox status of GSH/GSSG and glutathione. Studies have shown that CNMs, such as single-walled carbon nanotubes (SWCNTs), can increase the total flavonoid content in plants subjected to biotic stress, as well as in in vitro crops [76,77]. 

The control (T0) presented the highest concentration of phenols in the first sampling (Figure 6C), which may be linked to the fact that during the initial stages of growth, plants are actively building their defense system to protect themselves from various threats, in addition to being able to participate as growth promoters. In addition, the increase in the synthesis of phenols observed in the first sampling may be due to an adaptive response that guarantees an adequate antioxidant defense while the plant establishes and develops [78]. Although an increase in the average content of phenols was not observed during the three samplings, in the third sampling a significant increase was found in healthy and inoculated plants treated with NMs (Figure 6C). Specifically, an increase of 140% in the phenol content was observed in the plants treated with Gf compared with the negative control (T0); this was followed by the Gf + Fol treatment, which increased by 38.4% compared with the positive control, inoculated (Fol) (Figure 6C). It is suggested that this increase in the phenol content could be due to a greater decrease in the severity of the disease in the Gf + Fol treatment (Figure 1B), since previous studies have linked a high phenol content with the induction of resistance in tomato plants and a decrease in severity in plants infected by Fol [79,80].

The ROS-neutralizing enzyme system is composed of several enzymes, including ascorbate peroxidase, catalase (CAT), glutathione reductase (GR), superoxide dismutase (SOD), dehydroascorbate reductase (DHAR), glutathione-S-transferase (GST), and glutathione peroxidase (GPX) [65]. On average, of the three samplings in this study, the PAL enzyme activity increased in all treatments compared with the control and Fol (Figure 7A). In addition, the CAT enzyme stood out with a significant increase in GfCu and Gf treatments compared with control and GfCu + Fol and Gf + Fol compared with Fol (Figure 7B), while the activity of the GPX enzyme only increased in the treatments with functionalized graphene in healthy and inoculated plants compared with the negative control and Fol (Figure 7C). PAL enzyme activity is crucial for the synthesis of a wide variety of phenolic compounds in plants [81]. On the other hand, CAT is known to convert H_2_O_2_ into water and oxygen efficiently in cells exposed to environmental stress. This enzyme is found at major H_2_O_2_ production sites in the cellular environment of higher plants (such as peroxisomes, mitochondria, cytosols, and chloroplasts), and modulation of H_2_O_2_ by different catalase isozymes in specific cells or organelles interferes with H_2_O_2_ transduction signals in plants, suggesting that it plays an important role in adaptation to stress [82]. APX/GPX expression is related to the metabolic state of cells and these two enzymes are thought to work together in several metabolic pathways, including antioxidant metabolites and secondary metabolites, redox homeostasis, stress adaptation, and photosynthesis/respiration [83].

In previous studies, it has been shown that the application of phenylalanine-functionalized carbon nanotubes (f-MWCNT) in the induction of callus of basil (*Ocimum basilicum* L.) increases the activity of catalase, and this activity increases with the increase in the concentration of functionalized and non-functionalized carbon nanotubes. Furthermore, the highest activity of PPO, POD, and individual phenolic compounds was observed at specific concentrations of f-MWCNT and pristine MWCNT [84]. Graphene oxide (GO) has also been found to increase the activities of oxidative stress enzymes, such as CAT, POD, and SOD, in apple plants (*Malus domestica*), relative to controls [85]. In another study, wheat seedlings grown from seeds treated with polyhydroxyfullerene (PHF) showed recovery in root and shoot growth under salinity. This recovery was related to lower levels of MDA and H_2_O_2_ content and higher antioxidant activities of CAT, POD, and APX enzymes under salinity stress [63]. Chauhan et al. [86] found that PAL activity in the shoots of rice plants treated with Cu-NPs increased by 2- to 3-fold, suggesting that these nanoparticles possess potential in both controlling *Xanthomnas oryzae* and improving rice growth.

## 4. Materials and Methods

### 4.1. Crop Development

A greenhouse tomato crop was established using tomato seeds “El Cid F1” variety (Harris moran, Davis, CA, USA), of the saladette type and indeterminate growth. The transplant was carried out in 10 L black polyethylene containers in a mixture of peat moss-perlite substrate in a 1:1 ratio. For plant nutrition, Steiner solution was used [87]. Plants were managed on a single stem and developed for 22 weeks after transplant (WAT).

### 4.2. Treatments

**Figure 8:**
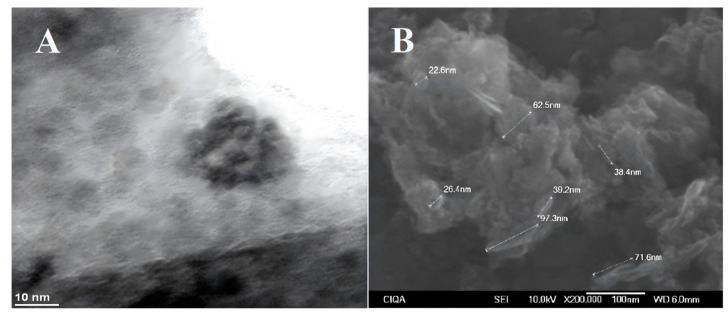

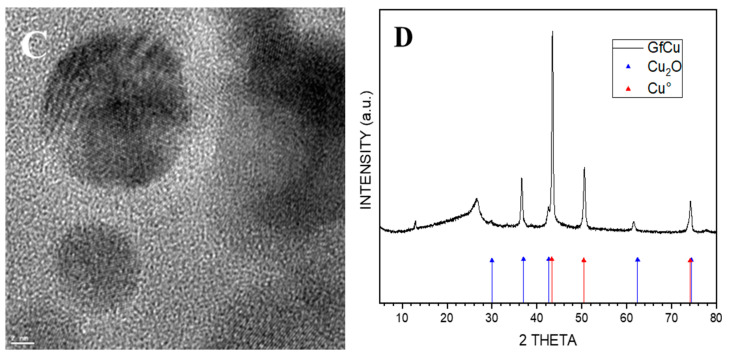
Cu-NPs (**A**) and functionalized graphene (**B**) obtained using scanning electron microscopy, HR-TEM of Cu-NPs (**C**), and XRD diffractogram of functionalized graphene (GfCu) (**D**).

Multilayer graphene nanoplatelets (GNP) with a diameter of 2 µm, a thickness of 8 to 12 nm, and a purity of 97% (Cheap Tubes Inc., Cambridgeport, VT, USA) were used. The functionalization of the graphene nanoplatelets was obtained with 4-aminobutyric acid, following the method described in previous studies [88]. Functionalized graphene nanoplatelets (FGNP) contained a 34.5% modification with respect to non-functionalized GNPs. The formation of Cu-NPs in the hybrid nanocomposite was carried out following a procedure similar to that previously reported [89]. A total of 5 g of FGNP was sonicated in 150 mL of distilled water for 20 min, then added to the glass reactor previously degassed with a stream of nitrogen. Next, 4.24 g of CuSO_4_∙5H_2_O, dissolved in 25 mL of distilled water, was added and mixed with a mechanical stirrer for 15 min at 300 rpm. After the time had elapsed, 6.7 mL of hydrazine was added and the mixture was stirred for 1 h under N_2_. The product was separated with centrifugation, washed with distilled water and ethanol, and finally dried at 90 °C in a vacuum oven. The Graphene–Cu hybrid nanocomposite contained 26.7% Cu-NPs determined with TGA [90]; the analysis using inductive coupled plasma (ICP) presented a similar value of 25.2%. Scanning electron microscopy (SEM) (Scanning Electron Microscopy model JEOL JSM-7401F.) analysis showed an average particle size of 6.0 nm (Figure 8A) and some platelet-like morphologies with different lengths (22–97 nm) (Figure 8B). The X-Ray diffraction (XDR) spectrum exhibited a broad peak located at 2θ = 26.67°, corresponding to graphene, and three reflections located at 2θ = 43.4°, 50.5°, and 74.0°, attributed to the (111), (200), and (220) crystal planes of the metallic copper. The peaks with 2θ values of 29.60°, 36.52°, 42.44°, 61.54°, 73.69°, and 77.61° corresponded to the crystal planes of 110, 111, 200, 220, 311, and 222 of crystalline Cu_2_O, respectively. The diffraction patterns (JCPDS No. 04-0836 and JCPDS No. 05-06667) corresponding to Cu and Cu_2_O, respectively, are shown in the lower part of the spectrum (Figure 8D).

The treatments applied were the following: (1) Negative control (T0), (2) Positive control inoculated with Fol, (3) Graphene–Cu nanocomposite (GfCu), (4) Graphene–Cu nanocomposite + Fol (GfCu + Fol), (5) functionalized graphene (Gf), and (6) functionalized graphene + Fol (Gf + Fol). Treatments with NMs were applied directly to the soil, 10 mL at a concentration of 100 mg L^−1^ at one-week intervals, starting at the transplant, with a total of 15 applications. 

### 4.3. Inoculation of F. oxysporum f. sp. lycopersici 

The spores of *F. oxysporum* f. sp. *lycopersici* were produced at 29 °C for 15 days in Petri dishes with potato destroxa agar (PDA) medium supplemented with ampicillin (100 mg L^−1^). The plants corresponding to the treatments with Fol were inoculated two weeks after transplantation with a spore suspension at a concentration of 1 × 10^8^ mL^−1^. In the first inoculation, 3 mL of conidial suspension was applied directly into the substrate of each plant. A second inoculation was performed one month later, applying 6 mL of 1 × 10^8^ mL^−1^ spore suspension. 

The incidence was determined considering the number of plants dead or with symptoms of the disease with respect to the total number of plants per repetition. The severity of tomato plants on foliage was rated on a scale of 0 to 4 as described by Grattidge and O’Brien [91], to evaluate Fusarium wilt, which was measured using a range of: 0 = Without visible symptoms of disease; 1 = Slight wilt, similar to lack of water; 2 = Similar to grade 1 + yellow or dry leaves, in less than 50% of the foliage; 3 = Similar to grade 1 + yellow or dry leaves on 50% or more of the foliage; and 4 = Plants completely wilted. These variables were determined from the inoculation and during the development time of the tomato crop.

### 4.4. Agronomical Parameters

At 26 (SDT) the culture was removed and the fresh biomass was quantified, as well as the dry biomass after drying it at 80 °C until presenting a constant weight. Moreover, plant height, stem diameter, number of clusters, number of leaves, number of fruits per plant, and fruit yield were determined.

### 4.5. Physiological Variables

Leaf water potential (Ψ_h_) was determined with a Scholander pressure pump (Soil moisture Equipment Corp^®^., Mod. 3115, Goleta, CA, USA) according to the methodology of Scholander et al. [92]. Assessments were performed beginning at 6 (SDT) at two-week intervals. Measurements were made some time before sunrise on fully developed leaves from each treatment and each replicate.

The efficiency of photosystem II (PSII) was assessed at 6 (SDT) at two-week intervals, using a fluorometer (Chl-fluorescence Analyzer, Yaxin-1162, Beijing Yaxinliyi Science and Technology Co., Ltd., Beijing, China). Basal fluorescence was determined (F0), QA reduction (Fj), electron transfer from QA to QB (Fi), and P fluorescence corresponding to PQ reduction (Fm, maximum fluorescence). Plants were dark-adapted for 30 min after dark, before measurement. With these variables, the maximum quantum yield for primary photochemistry was determined when all PSII reaction centers were oxidized or “open” (Fv/Fm: where Fv is the variable fluorescence = [Fm−F0]). 

### 4.6. Biochemical Variables

Three samples were taken for biochemical analysis. Sampling one was carried out at 6 (WAT), sampling two at 10 (WAT), and the third sampling at 14 (WAT) considering fully developed young leaves. For sampling, the fully expanded young leaves (third or fourth leaf) were collected and placed on ice for later storage at −20 °C. The samples were lyophilized and macerated until a fine powder was obtained.

The contents of chlorophylls a and b (mg g^−1^ DW) were determined according to the method of Nagata and Yamashita [93]. The lyophilized sample (10 mg) was mixed with 2 mL of hexane/acetone (3:2). Subsequently, the samples were subjected to an ultrasonic bath for 5 min. They were then centrifuged at 15,000× *g* for 10 min at 4 °C. The supernatant was removed and the absorbance was read at 645 and 663 nm using a spectrophotometer. The obtained values were used in Equations (1) and (2) to calculate the chlorophyll content.

(1) $$Chlorophyll\;A\;=\;0.999\;*\;A_{663}-0.0989{\;*\;A}_{645}$$

(2) $$Chlorophyll\;B\;=0.328\;*\;A_{663}+1.77{\;*\;A}_{645}$$

Ascorbic acid (mg 100 g^−1^ 1 DW) was determined by means of spectrophotometry. Briefly, 10 mg of lyophilized tissue was extracted with 1 mL of 1% metaphosphoric acid (HPO_3_) and filtered with Whatman No. 1 filter paper. For quantification, 200 μL of extract was taken and mixed with 1800 μL of 2,6 dichlorophenol indophenol (100 mM), with absorbance measured at 515 nm on a UV-Vis spectrophotometer (UNICO Spectrophotometer, Model UV2150, Dayton, NJ, USA) [94].

Glutathione (GSH) (mmol 100 g^−1^ DW) was determined using the method of Xue et al. [95] using 5,5-dithio-bis-2 nitrobenzoic acid (DTNB) reaction. In total, 0.480 mL of the extract, 2.2 mL of sodium dibasic phosphate (Na_2_HPO_4_ at 0.32 M), and 0.32 mL of the DTNB dye (1 mM) were placed in a test tube. Then, the suspension was vortexed and read on an UV-Vis spectrophotometer (UNICO Spectrophotometer Model UV2150, Dayton, NJ, USA) at 412 nm using a quartz cell.

The content of total phenols (mmol 100 g^−1^ DW) was obtained according to Yu and Dahlgren [96]. In total, 100 mg of lyophilized tissue was extracted with 1 mL of a water/acetone solution (1:1) and the mixture was homogenized for 30 s. The sample tubes were centrifuged at 17,500× *g* for 10 min at 4 °C. Then, 18 μL of the supernatant, 70 μL of the Folin–Ciocalteu reagent, and 175 μL of 20% sodium carbonate (Na_2_CO_3_) were placed in a test tube, and 1750 μL of distilled water was added. The samples were placed in a water bath at 45 °C for 30 min. Finally, the reading was taken at a wavelength of 750 nm on the UV-Vis spectrophotometer (UNICO Spectrophotometer, Model UV2150, Dayton, NJ, USA). Total phenols were expressed in mg EQ of gallic acid per gram of DW.

The flavonoid content (mmol 100 g^−1^ DW) was determined according to Arvouet-Grand et al. [97]. For the extraction, 20 mg of lyophilized tissue was placed in a test tube to which 2 mL of reactive-grade methanol was added, and this was homogenized for 30 s. The mixture was filtered using Whatman No. 1 paper. For the quantification, 1 mL of the extract and 1 mL of 2% methanolic aluminum trichloride (AlCl_3_) solution were added to a test tube and allowed to stand for 20 min in darkness. The reading was taken at a wavelength of 415 nm on a UV-Vis spectrophotometer (UNICO Spectrophotometer, Model UV2150, Dayton, NJ, USA). The results were expressed in mg EQ of quercetin per gram of DW.

The anthocyanin content (mmol 100 g^−1^ DW) was determined according to the methodology of Lee et al. [98], and the results were expressed as mg cyanidin-3-glucoside equivalents per gram of DW. In total, 50 mg of lyophilized sample was homogenized with 2000 µL of methanol containing 1% HCl. The homogenate was centrifuged at 8000× *g* for 10 min at 4 °C. The reaction mixture consisted of 2 phases: in phase 1, 400 µL of extract was mixed with 1600 µL of 0.025 M potassium chloride (pH 1.0); in phase 2, 400 µL of extract was mixed with 1600 µL of 0.4 M sodium acetate chloride (pH 4.5). The absorbance of both samples was read at 520 and 700 nm using methanol as the blank. The anthocyanin content was determined using the following equation:

(3) $$\frac{A\times MW\times DF\times 10^{3}}{\varepsilon \times 1}$$

where $A=\left(A_{520nm}-A_{700nm}\right)pH\;1.0-\left(A_{520nm}-A_{700nm}\right)pH\;4.5$; *MW* (molecular weight) = 449.2 g mol^−1^ for cyanidin-3-glucoside; *DF* = dilution factor established in D; 1 = path length in cm; *ε* = 26,900 molar extinction coefficient, in $L\times {mol}^{-1}\times {cm}^{-1}$, for cyanidin-3-glucoside; and 10^3^ = factor for conversion from g to mg.

The glutathione peroxidase (EC 1.11.1.9) [U per gram of proteins (U g^−1^ P), where U is equal to the mmol equivalent of reduced glutathione (GSH) per milliliter per minute] was determined using the method of Xue et al. [95]. A mix of 200 μL of extract, 400 μL of GSH (0.1 mM), and 200 μL of Na_2_HPO_4_ (0.067 M) was placed in a test tube. The mixture was preheated in a water bath at 25 °C for 5 min, then 200 μL of H_2_O_2_ (1.3 mM) was added to start the catalytic reaction for 10 min at a temperature of 26 °C. The reaction was stopped by the addition of 1 mL of 1% trichloroacetic acid. The mixture was placed in an ice bath for 30 min and then centrifuged at 1000× *g* for 10 min at 4 °C. To assess the glutathione peroxidase, 480 μL of the supernatant, 2.2 mL of Na_2_HPO_4_ (0.32 M), and 320 μL of 5,5-dithio-bis-2-nitrobenzoic acid dye (DTNB) of 1 mM were placed in a test tube. The absorbance was measured on an UV-Vis spectrophotometer (UNICO Spectrophotometer, Model UV2150, Dayton, NJ, USA) at 412 nm with a quartz cell.

The catalase (EC 1.11.1.6) (U g^−1^ P, where U is equal to the mmol equivalent of H_2_O_2_ consumed per milliliter per minute) was quantified with the method of Dhindsa et al. [99]. The measurement was carried out in two moments [at time 0 (T0) and at time 1 (T1)]. At T0, 200 μL of extract and 2 mL of H_2_O_2_ (100 mM) were added to a test tube and vortexed for 30 s. The absorbance was then measured on a UV-Vis spectrophotometer (UNICO Spectrophotometer, Model UV2150, Dayton, NJ, USA) with a quartz cell at 270 nm. The measurement at T1 was taken after 60 s of reaction. The determination of catalase was based on the quantification of the oxidation rate of H_2_O_2_ using absorbance difference (T0–T1).

The phenylalanine ammonia lyase (EC 4.3.1.5) was determined according to Sykłowska-Baranek et al. [100], and the results expressed as U per gram of proteins (U g^−1^ 1 P), where U is equal to μmol equivalent of trans cinnamic acid per milliliter per minute. A total of 0.1 mL of the enzymatic extract was taken, and 0.9 mL of L-phenylalanine (6 mM) was added. After 30 min of incubation at 40 °C, the reaction was stopped with 0.25 mL of 5 N HCl. The samples were placed in an ice bath, and 5 mL of distilled water was added. The absorbance was determined at 290 nm on a UV-Vis spectrophotometer (UNICO Spectrophotometer, Model UV2150, Dayton, NJ, USA).

Hydrogen peroxide (H_2_O_2_) was assessed according to the methodology described by Velikova et al. [101] and expressed as μmol g^−1^ of DW. In total, 10 mg of lyophilized sample was homogenized with 1000 µL of cold trichloroacetic acid (0.1%). The homogenate was centrifuged at 12,000× *g* for 15 min and 250 µL of the supernatant was added to 750 µL of potassium phosphate buffer (10 mM) (pH 7.0) and 1000 µL of potassium iodide (1 M). The absorbance of the supernatant was read at 390 nm. The content of H_2_O_2_ was given on a standard.

### 4.7. Statistical Analysis

The experiment was established under a Latin square design considering six repetitions per treatment. For the agronomic and biochemical variables, an analysis of variance and a Fisher’s least significant difference test (α = 0.05) were performed using InfoStat software (v2020) (Universidad Nacional de Córdoba, Córdoba, Argentina). For the evaluation of incidence, severity, and physiological variables, a multivariate analysis of variance (MANOVA) and a Hotelling test (α = 0.05) were performed.

## 5. Conclusions

The application of Graphene–Cu nanocomposites and functionalized graphene in tomato plants inoculated with Fol succeeded in delaying the incidence of tomato “vascular wilt” disease and reducing its severity. This translated into an increase in the content of photosynthetic pigments and an increase in fruit production compared with Fol, and this can potentially decrease the negative effects of fusarium wilt on the fruit yield of tomato crops. In addition, these nanocomposites improved the antioxidant system by increasing the content of GSH, flavonoids, anthocyanins, and the activity of GPX, PAL, and CAT enzymes. 

Both photosynthetic pigments and secondary metabolites played a crucial role in neutralizing ROS, resulting in improved water stress response and photosystem II (Fv/Fm) efficiency. These metabolites are associated with the tolerance of plants against biotic stress, indicating that Graphene–Cu and functionalized graphene nanocomposites could induce tolerance against Fol indirectly through the synthesis of secondary metabolites and antioxidant defense system.

The results obtained in this work show that the use of Graphene–Cu nanocomposites can be very useful for the management of pathogens (such as Fol) that attack agricultural crops. Additionally, they can be an interesting option to be applied in the medium term, either to complement the management of pathogens with currently available chemical products, or even to develop new products that may be more efficient. However, it is necessary to carry out more in-depth studies considering omics sciences, and with a greater number of pathogens and crops to obtain the necessary information that allows us to define the optimal concentrations in specific application routes. In addition, it is important to study the possible impacts of nanocomposites on other types of organisms, such as beneficial soil organisms, and the residuality or transfer capacity in trophic chains.

## Data Availability

Not applicable.
